# Management of cushing’s syndrome in patients with adrenocortical cancer: state of the art and future perspectives

**DOI:** 10.1007/s11154-025-09989-y

**Published:** 2025-07-30

**Authors:** Valentina Guarnotta, Antonio Stigliano, Massimo Terzolo, Giorgio Arnaldi

**Affiliations:** 1 Unit of Endocrinology, Department of Health Promotion, Mother and Child Care, Internal Medicine and Medical Specialties, Policlinico Paolo Giaccone, Università degli studi di Palermo, Piazza delle Cliniche 2, 90127 Palermo, Italy; 2 Endocrinology, Department of Clinical and Molecular Medicine, Faculty of Medicine and Psychology, Sant’ Andrea University Hospital, Sapienza University of Rome, 00189 Rome, Italy; 3 Internal Medicine, Department of Clinical and Biological Sciences, S. Luigi Gonzaga Hospital, University of Turin, 10043 Orbassano, Italy

**Keywords:** Cushing’s syndrome, Adrenocortical carcinoma/cancer, Steroidogenesis inhibitors, Cortisol-secreting, Glucocorticoid excess

## Abstract

Adrenocortical cancers (ACCs) are rare tumours, with up to 50% of cases associated with hypercortisolism. Cortisol-secreting ACCs are characterized by a worse prognosis, and in these patients, the normalization of hypercortisolism is mandatory and requires an urgent approach to avoid complications related to glucocorticoid excess. Clinical and biochemical parameters, including hormonal values, can be used to define cortisol normalization. However, in patients on concomitant mitotane treatment, serum cortisol and ACTH levels may be falsely altered and thus unreliable for defining cortisol normalization. Adrenal steroidogenesis inhibitors, alone or in combination, are the first-line treatment for severe hypercortisolism in ACC due to their rapid action, efficacy, and safety profile. Mitotane is the cornerstone of ACC treatment in both adjuvant and advanced settings. Similarly, glucocorticoid receptor antagonists also have a rapid onset of action, but their use is limited by challenges in monitoring efficacy and safety. This review aims to address the critical aspects of managing cortisol-secreting ACC, including the definition of hypercortisolism control, current therapeutic approaches and future perspectives for ACC, with a focus to the potential role of immune checkpoint inhibitors.

## Background

Adrenocortical cancer (ACC) is a rare tumour that arises from adrenal cortex. Its incidence is on average 1–2 case per million persons per year and affects more frequently women (55–60% of cases) [1]. The incidence follows a bimodal age distribution, with a first peak in childhood and a second plateau between 40 and 50 years of age. It is generally a sporadic tumour, even though it can be also part of a hereditary syndrome including the Li Fraumeni (*TP53*), multiple endocrine neoplasia type 1 (MEN 1), Lynch syndrome (*MSH2*, *MLH1*, *PMS2*, *MSH6*, *EPCAM*), Carney complex (*PRKAR1A*), type 1 neuro fibromatosis (*MEN1*), familial adenomatous polyposis coli (*APC*) and Beckwith–Wiedeman (*IGF2* locus) [2].

ACC has a complex pathogenesis due to chromosomal aberrations, epigenetic and genetic mutations [3]. It is an aggressive tumour with a poor 5-year prognosis ranging from 16 to 47%, strongly influenced by age, tumour stage, hormonal hypersecretion, mitotic rate, tumour grade and surgical resection margins [4–6].

ACC can present as a non-functioning tumour showing symptoms as back or abdominal pain, nausea, vomiting, slight fever, weight loss or as a hormone-secreting tumour. Hormonal hypersecretion is very frequent in patients with ACC although the degree of secretion is highly variable and consequently the clinical consequences are also different [7, 8]. It is estimated that more than 50% of ACC are hyperfunctioning and among them at least 60% of cases are characterized by cortisol hypersecretion alone or combined with adrenal androgens (25%, mainly testosterone). Rare is aldosterone secretion (7–8%) and extremely rare oestrogen secretion (1–2%) [9].

## Adrenocortical cancer and severe hypercortisolism: clinical characteristics and prognosis

Cortisol-secreting ACC are characterized by worse prognosis, higher recurrence rates, and worse survival than non-functioning ACCs [7].

Clinical presentation can be different depending on the degree and the sudden onset of hypercortisolism [7].

In patients with mild hypercortisolism the onset of clinical symptoms can be slower than severe hypercortisolism and include facial plethora, easy bruising, weight gain and diabetes mellitus.

In severe hypercortisolism the clinical onset of the disease is very rapid and life-threatening. It can manifest with severe and resistant hypertension often associated with a severe hypokaliemia that frequently requires an immediate hospitalization. Hypokaliemia is generally due to the glucocorticoid excess that triggers mineralcorticoid receptors inactivating the capacity of 11beta-hydroxysteroid dehydrogenase isoenzyme 2 (HSD11B2). ACC patients with severe hypercortisolism can also manifest with deep venous thrombosis, pulmonary embolism, sepsis, acute psychosis and severe hyperglycaemia.

Cortisol hypersecretion is generally viewed as an independent worse prognostic parameter of ACC [2, 10] and it is generally associated to increased mortality and morbidity [11]. Currently, there are few studies which evaluated whether hypercortisolism control is associated to a better overall survival and whether the effects of hypercortisolism control could be attributed to anti-neoplastic therapy [12].

Further, a recent retrospective study showed that 4 out of 74 patients with ACC died before any specific cancer specific treatment for lethal complications of hypercortisolism, but not for tumoral progression [13].

In a multicentric study conducted on 524 patients with ACC (ENSAT stage 1-2-3), hypercortisolism was confirmed to be a negative prognostic factor, in terms of relapse-free survival and overall survival, even though cortisol levels were not associated with mitotic index [5]. This finding could support the hypothesis that in patients with hypercortisolism other mechanisms could correlate the cortisol excess with the tumour aggressiveness. Maybe, the decrease of overall survival in these patients could be attributed to the effects of cortisol excess in increasing mortality, and to the suppressive effects of hypercortisolism on immune system, rather than a direct effect.

According to most recent guidelines the normalization of hormonal excess represents a priority in the management of patients with hormone-secreting ACC and medical therapy is strongly recommended to obtain the hormonal control [14].

Although there are no data on the association between hypercortisolism control and overall survival, it is conceivable that the control of thromboembolic, metabolic and cardiovascular risk may be correlated to better outcome.

## Assessing and monitoring hypercortisolism control during medical therapy in ACC

The clinical and biochemical criteria to define control of hypercortisolism are not different from those used in other forms of Cushing’s syndrome (CS) even though the complexity of the clinical picture often requires special considerations and precautions [15]. Certainly, patients should be managed in referral centers by physicians with extensive experience, generally defined as the management of at least 5–10 new cases of ACC per year.

The treatment of hypercortisolism is aimed at improving the clinical and biochemical parameters which then allow tumour-directed treatments (surgery or chemotherapy). The same antineoplastic treatment could then influence hormonal secretion and therefore could contribute to the clinical benefit.

There are few studies that have analyzed in detail the effects obtained from the control of hypercortisolism compared to therapy aimed at treating the tumor. The use of mitotane does not allow us to distinguish with certainty the two actions given by the mechanism of action of this drug.

Clinical parameters include blood pressure, weight and CS symptoms and signs. A decrease in the number of anti-hypertensive drugs, anti-diabetic agents, weight loss and improvement in phenotypic characteristics are useful markers of hypercortisolism control. Notably, changes in blood pressure are very rapid and for this reason blood pressure lowering can be considered as an early predictor of hypercortisolism control. Biochemical parameters include serum potassium level, coagulation parameters and glycaemic values whose changes occur rapidly and are not affected by a potential mitotane therapy.

Further, also hormonal parameters could be evaluated including urinary free cortisol (UFC), early morning serum and salivary cortisol and ACTH, even though in case of concomitant administration of mitotane, some of these values may be unreliable. Indeed, UFC may be falsely elevated as mitotane alters steroid clearance and increased catabolism [16]. Salivary cortisol is not affected by cortisol binding globulin (CBG) alterations and might reflects the free serum cortisol and may enable a more accurate evaluation of cortisol secretion [17]. By contrast, mitotane treatment could affect serum cortisol due to its effect on the increase in CBG and ACTH levels by inhibitory effect. In addition, some steroidogenesis inhibitors, as metyrapone and osilodrostat, can cause increase in 11-deoxycortisol, which can cross-react in many immunoassays for serum and urinary cortisol, resulting in apparently high cortisol values and potentially masking biochemical hypoadrenalism [18].

In clinical practice, monitoring should be performed at baseline, before starting medical therapy, and then at regular intervals based on the clinical status and pharmacologic treatment used. Initially, assessments every 2–4 weeks may be appropriate to guide dose titration and evaluate early treatment effects. Once a biochemical and clinical response is achieved, the monitoring interval may be extended to every 2–3 months.

Particular attention should be paid to potential rapid changes in blood pressure, glycaemia and potassium levels, which may require prompt therapeutic adjustment. Hormonal parameters (urinary free, serum and salivary cortisol levels) should be interpreted with caution during mitotane treatment. These parameters should ideally be assessed using mass spectrometry, which minimizes cross-reactivity, provides more accurate cortisol measurements, and is less influenced by CBG variations [19].

## Mitotane, steroidogenesis inhibitors and combined treatment

The drugs, either alone or in combination, are the same as those used in the treatment of severe hypercortisolism [18]. In the case of mild hypercortisolism, however, the use of mitotane alone may be sufficient although its efficacy is often delayed by several days/weeks.

As mentioned, in patients with severe hypercortisolism a rapid control should be obtained. Although surgery represents the treatment of choice, sometimes it cannot be performed immediately. Adrenal directed drugs, steroidogenesis inhibitors, can instead effectively be used to manage rapidly severe hypercortisolism, either as monotherapy or in combination with other drugs including mitotane [14, 20].

Mitotane is the mainstay of treatment of ACC both as adjuvant treatment and in patients not candidates for surgery or with metastatic disease. The drug acts primarily on fasciculata and reticularis zonae, less on glomerular of adrenal gland reducing the glucocorticoids and 17-hydroxycorticosteroids synthesis, beyond to inhibit the expression of many enzymes involved in steroidogenesis [21]. Furthermore, this reduces cellular energy mechanisms by acting on the mitochondrial machinery [22]. For its characteristic mechanism of action, mitotane can be considered both an adrenolytic and a steroidogenesis inhibitor drug ensuring an adequate control of hypercortisolism. However, due to the necessity of attaining therapeutic levels its efficacy is delayed by several weeks with a slow effect. For this reason, mitotane alone is not recommended for the management of severe hypercortisolism, while it should be used for mild hypercortisolism.

Currently, a comparative study of the efficacy of steroidogenesis inhibitors in the management of severe hypercortisolism in ACC is lacking, while there are studies evaluating the efficacy of single steroidogenesis inhibitors and glucocorticoid receptor antagonists.

Ketoconazole is an imidazole derivative anti-fungal compound that inhibits adrenal steroidogenesis by acting at several cytochrome P450 steroidogenic enzymes. Adverse events include gastrointestinal symptoms and headache, and in the long-term, irregular menses in females and decreased libido and gynaecomastia in males respectively. In addition, hepatotoxicity could develop. Despite its clinical efficacy, characterized also by a rapid action in controlling severe hypercortisolism [23], it interacts with CYP3A4 that is also the substrate of interaction of mitotane. The mechanism of actions on CYP3A4 of mitotane and ketoconazole are different [24]. Indeed, mitotane is a CYP3A4 inducer, while ketoconazole is a CYP3A4 inhibitor. However, in the overall context it is difficult to predict the effects of their combination and a therapeutic drug monitoring should be done in a hospital setting. Ketoconazole should be avoided at the start of mitotane because for the reasons stated above it will be difficult to attribute hepatotoxicity to one or the other drug. It should, however, be noted that for adequate oral absorption, ketoconazole requires an acidic gastric environment and the suggested dose to treat hypercortisolism is 400–1200 mg/day.

Corcuff et al. reported 22 patients with severe hypercortisolism, 14 with ectopic Cushing’s syndrome and 8 with ACC, treated with a combination of ketoconazole and metyrapone [25]. All patients had hypertension, 6 out 8 had hypokalaemia, 3 out 8 had diabetes mellitus. Ketoconazole was started with a variable dose ranging from 600 to 1200 mg/day, while metyrapone had a starting dose ranging from 750 to 4500 mg/day. In 5 out of 8 patients mitotane was started immediately in combination with metyrapone and ketoconazole. UFC was normalized after 1 week in 5 out 8 patients. After 1 month blood pressure, glycaemia and potassium values were also improved in the most of patients. Only a patient interrupted ketoconazole due to liver toxicity.

Levoketoconazole is the 2 S,4R stereoisomer of ketoconazole available as an immediate-release tablet containing 150 mg compound. Levoketoconazole inhibits adrenal steroidogenesis more potently than ketoconazole but its hepatic exposure is less extensive. The therapeutic dose is 150–600 mg twice-daily and reduce safety concerns on QT interval prolongation, hepatotoxicity and CYP7A1-mediated drug-drug interactions. FDA recently approved levoketoconazole for the treatment of CS in adults who are not eligible or have failed surgery. However, to our knowledge, there are no studies on levoketoconazole treatment of hypercortisolism associated with ACC.

Metyrapone is an 11-beta-hydroxylase inhibitor blocking the conversion of 11-deoxycortisol in cortisol. This drug is available as an immediate-release capsule containing 250 mg compound, after oral ingestion, is absorbed rapidly but due to its high inter-individual variability in pharmacokinetics and short half-life it requires four-to-six daily administrations at doses ranging from 500 to 6000 mg/day. It is characterized by a rapid onset, within 24–72 h and high efficacy [26]. Its metabolism and elimination are not influenced by mitotane and for this reason the combination of these two drugs can be considered safe [27]. Metyrapone efficacy in severe hypercortisolism is scarcely documented although in ENSAT ACC Guideline [14] this drug was considered the first therapeutic choice for the management of patients with advanced ACC and severe CS. Indeed, there are no studies on severe hypercortisolism treated with metyrapone alone, since in most studies a combination therapy was employed, rather than a monotherapy.

Claps et al. reported 3 male patients with cortisol-secreting ACCs, two with a metastatic form and the other one with a locally advanced form [27]. Only one out of 3 had a severe hypercortisolism. Combined chemotherapy, etoposide + doxorubicin and platinum, mitotane and metyrapone were used. Mitotane was started at dose of 3000 mg/daily and metyrapone at the dose of 750 mg/daily (that corresponded at the maximum dose). After 4 weeks of treatment, the patients showed an improvement of clinical conditions combined with an increase in potassium value and a decrease in UFC. No significant change in serum cortisol was observed. The control of hypercortisolism was likely due to the synergistic effect of metyrapone and mitotane, which led to a rapid clinical improvement. At 12 weeks one patient had a minimal response and another one had a partial response, while the third patient had a progressive disease.

Osilodrostat is an oral inhibitor of adrenal 11β-hydroxylase and aldosterone synthase currently approved by FDA and EMA for the treatment of CS. It is well tolerated. The suggested initial dosage is 2 mg twice daily. Up-titration should be gradual and based on individual response (usually, cortisol levels and/or symptoms of adrenal insufficiency) and tolerability. The usual daily maintenance dose was 4–14 mg and the maximum dosage is 30 mg twice daily. Osilodrostat has been demonstrated to be efficacious in obtaining rapid serum cortisol and blood pressure reductions. In 2020, Haissaguerre et al. reported a case of severe hypercortisolism due to an ACC who interrupted ketoconazole for liver toxicity and was treated with the combination of osilodrostat and mitotane obtaining a normalization of cortisol values after 2 weeks [28]. In 2022, Tabarin et al. reported 7 cases of ACC treated with osilodrostat [12]. Mitotane was combined in 3 patients. Two had a previous treatment with metyrapone, which was interrupted due to the lack of efficacy. After two weeks a significant decrease in UFC was observed in 6 out of 7 patients. The mean doses were ranging from 4 to 40 mg/day, even though only 2 patients were treated with a dose less than 10 mg/day. No treatment escape was observed, even though the mean duration of treatment was quite short (14 weeks). In addition, the treatment was safe and well tolerated. One patient showed a worsening of hypokaliemia and 3 experienced adrenal insufficiency, which was adequately managed by hydrocortisone replacement therapy.

Etomidate is an anaesthetic agent with sedative-hypnotic activity. It has got also an adrenocortical inhibition action blocking the 11β-hydroxylase, CYP17A1 and cholesterol side-chain cleavage enzyme. It is generally used in an emergency setting in hospitalized patients, to rapidly treat hypercortisolism, due also to its parenteral administration and it is considered an adequate therapy for severe hypercortisolism [29]. Currently, there are only some case reports on the use of etomidate in cortisol-secreting ACC, confirming its successful effect in rapid normalization of cortisol levels and improvement in blood pressure [30–33]. Due to its characteristics, etomidate should be recommended in patients who need to reach a rapid control of hypercortisolism or cannot take oral therapy.

The above-mentioned studies are reported in Table 1.

**Table 1:** Studies on steroidogenesis inhibitors and glucocorticoid receptor antagonists for the treatment of cortisol-secreting ACCs

Authors	Drugs	Patients	Maximum dose	Starting dose	Stage of disease	Time of hypercortisolism control	Combination with mitotane	Parameters evaluated to define hypercortisolism control	Replacement hydrocortisone therapy
Claps et al.	Metyrapone	3	750 mg/day	750 mg/day	2 pts: IV stage 1 pt: III stage	4 weeks	Yes	Weight, K, UFC, ACTH, serum cortisol, glucose	No
Corcuff et al.	Metyrapone and ketoconazole	8	Ketoconazole 1200 mg/day Metyrapone 4500 mg/day	Ketoconazole 600–1200 mg/day Metyrapone 750–4500 mg/day	3 pts: Stage II 5 pts: Stage IV	1 week (5/8) 1 months (7/8)	Yes (5/8)	UFC, midnight cortisol, midnight ACTH, blood pressure, K, glucose	Yes (2/8)
Haissaguerre et al.	Osilodrostat	1	44 mg/day	5 mg/day	NA	2 weeks	Yes	Serum cortisol	Yes
Tabarin et al.	Osilodrostat	7	40 mg/day	2–20 mg/day	5pts: Stage IV 1 pt: Stage III 1 pt: Stage II	1 week (2/7) 1 month (1/7) 2 months (2/7) 3 months (2/7)	Yes (7/8)	UFC, serum cortisol, K, glucose, blood pressure, phenotypic characteristics	Yes (2/7)
Łebek-Szatańska et al.	Etomidate + ketoconazole	1	5 mg/h	2.5 mg at bolus and an infusion of 0.01–0.02 mg/kg/h (1–2 mg/h) etomidate + 1200 mg/day ketoconazole	Stage IV	4 days	No	Serum cortisol, glucose, K, blood pressure	Yes
Wan Muhamad Hatta et al.	Etomidate	1	42.8 mcg/kg/h	42.8 mcg/kg/h	Stage IV	7 days	Yes	Serum cortisol	No
Huang CJ et al.	Etomidate	1	2 mg/h	2 mg/h continuous infusion	Stage IV	2 days	No	Serum cortisol	No
Castinetti et al.	Mifepristone	12	2000 mg/day	400–1000 mg/day	Stage IV	1–6 months	Yes	Blood pressure, clinical signs, K,	Yes (2/12)

Abbreviations: pts: patients; UFC urinary free cortisol; K potassium

## “Block and replace” or “titration-to-normalization” treatment protocols

Generally, there are two treatment strategies based on patients’ clinical picture and biochemical characteristics: the “titration” or “normalization” regimen aims at obtaining control of hypercortisolism via gradual dose up-/down-titration, whereas the “block and replace”, which combines higher adrenostatic doses with glucocorticoid replacement.

The “titration to normalization” approach is commonly used in patients with mild or moderate hypercortisolism. It is based on the use of steroidogenesis inhibitors which could be initiated at low daily doses (e.g., ketoconazole 400–600 mg, metyrapone 500–750 mg, osilodrostat 4 mg, levoketoconazole 300 mg), progressively increased, to reach eucortisolism. This approach is generally slower but avoids adrenal insufficiency and can be effective.

**Fig. 1:**
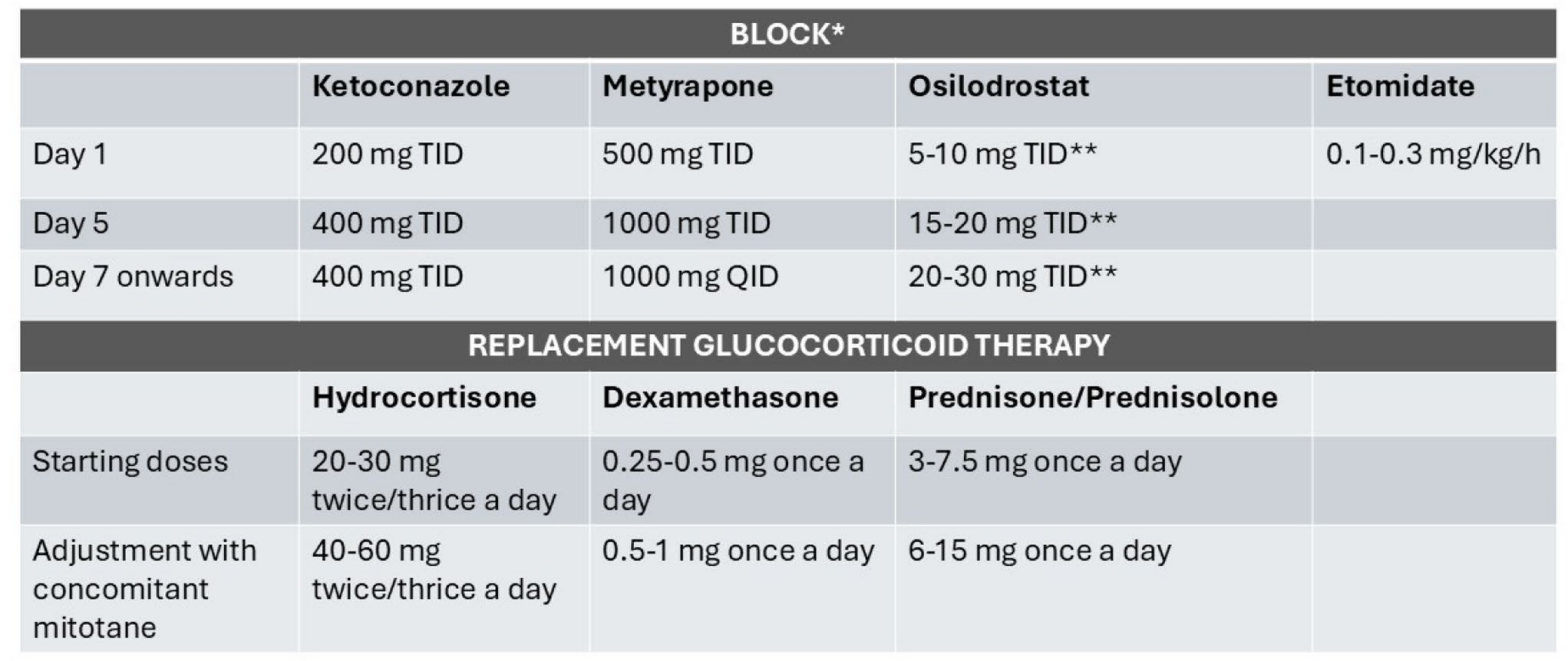
Personal expert opinion in medical management of “block and replace” scheme of treatment. *strict monitoring of the patient is required. **personal view

The “block and replace” regimen is used in severe hypercortisolism in order to obtain a rapid control of hypercortisolism and to avoid an alternation between over and underdosing [34]. However, block and replace regimen is more expensive and requires more tablets per day and a good patient’s compliance. A potential scheme of “block and replace” regimen could involve high doses of steroidogenesis inhibitors as reported by Corcuff et al. who reported mean starting doses of metyrapone of 2125 mg/day and ketoconazole of 900 mg/day [25]. Osilodrostat could be started at a dose of 5–10 mg twice daily to be increased in the following days up to 20–30 mg twice daily [34, 35]. Hydrocortisone could be started at same doses of adrenal insufficiency based on patients weight (0.12 mg/kg) [36] or body surface (10–12 mg/m^2^ daily) [37] (Fig. 1). However, in patients receiving mitotane, glucocorticoid dose adjustments are necessary.

Mitotane-induced CYP3A4 activation leads to rapid inactivation of more than 50% of administered hydrocortisone, requiring at least a doubling of the replacement dose to achieve glucocorticoid exposure comparable to that in individuals not receiving CYP3A4-inducing agents [38].

The risk of adrenal insufficiency and side effects (especially gastrointestinal and hepatic) must always be considered, especially when high doses are used. Patients need to be educated about symptoms and signs of adrenal insufficiency. All patients at risk for adrenal insufficiency need to be supplied with emergency medication and instructions.

Metyrapone and osilodrostat can lead to high blood pressure, hypokalemia, edema and androgens secondary to the accumulation of precursors with mineralocorticoid activity such as 11-deoxycortisol and 11-deoxycorticosterone.

Hypokalemia depending on its severity, is treated with oral potassium or parenteral administration. Spironolactone and other potassium-sparing drugs can be use highly effective, monitoring of serum potassium to the risk of hyperkalemia.

With regard to etomidate, treatment protocols include high-doses (0.5-1 mg/kg/h) which, generally, require combined treatment with intravenous hydrocortisone, to avoid adrenal insufficiency, or low-doses (0.04–0.05 mg/kg/h) which induce partial suppression [31, 39].

In emergency settings, an intravenous bolus of 3–5 mg of etomidate administered over 30–60 s followed by infusion rate of 0.02–0.10 mg/kg/h, has been shown to rapidly suppress cortisol production in most patients [40, 41].

**Fig. 2:**
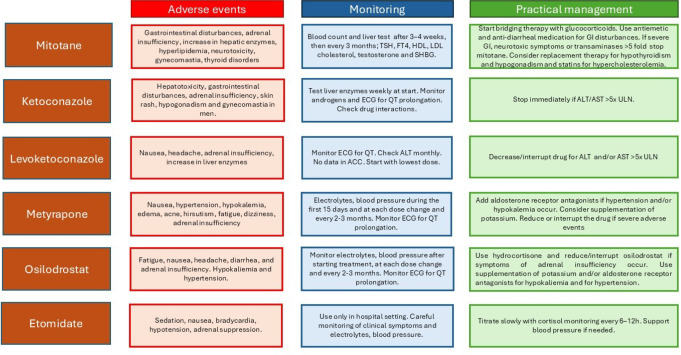
Adverse events, monitoring, and therapeutic management of mitotane and steroidogenesis inhibitors

**Fig. 3:**
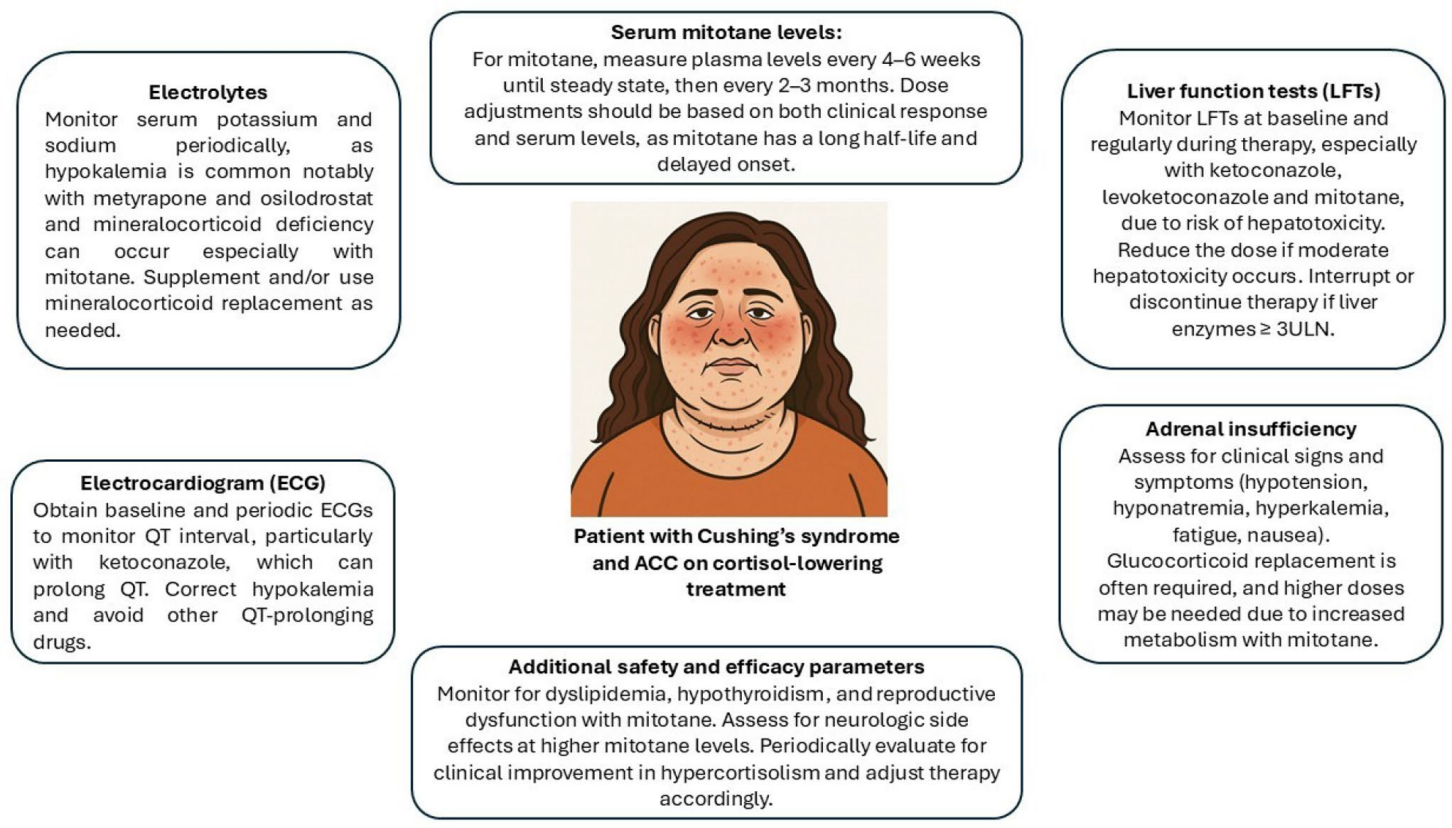
Overview of adverse event monitoring and management in a patient with adrenocortical carcinoma (ACC) and Cushing’s syndrome receiving cortisol-lowering therapy (picture generated by artificial intelligence)

The management of cortisol-lowering treatment adverse events is reported in Figs. 2 and 3.

## Glucocorticoid receptor antagonists

Mifepristone is a glucocorticoid receptor antagonist with an 18-fold higher affinity to the glucocorticoid receptor than cortisol, with anti-androgen and anti-progestin effects. Mifepristone is approved by the US FDA for the treatment of hyperglycemia secondary to CS in patients with disease recurrence or when they are not amenable to surgery. It is characterized by rapid action and efficacy in severe hypercortisolism. However, due to its mechanism of action, its efficacy can be monitored only by glucose, weight and blood pressure values. Potassium should be monitored due to the risk of adrenal insufficiency. Castinetti et al. reported 11 patients with ACC and hypercortisolism who were previously treated with other therapies including surgery, chemotherapy and mitotane [42]. Eight patients had also a previous treatment with other anti-cortisol drugs (ketoconazole, metyrapone and etomidate). In eight patients there was an improvement in hypercortisolism signs within the first month. The median dose was 400 mg/day (200–1000 mg/day) and a median duration of treatment of 2 months (5 days to 6 months). Mifepristone was associated with improvement of psychiatric symptoms, blood pressure and diabetes mellitus, while hypokalaemia was worsened. In 8 patients a tumour progression was observed and 3 stopped the treatment for lack of efficacy and hypokalaemia worsening. In the end 2 patients experienced adrenal insufficiency.

### Future directions

Relacorilant is a highly selective glucocorticoid modulator, that competitively antagonizes cortisol activity, without anti-progestin effects. It is an investigational product and it has not yet been approved for the treatment of CS, although it has demonstrated promising results (patients with ACC were excluded in this study) [43]. Currently, due to the novelty of this drug, only a clinical trial has been performed on its use in ACC. This trial evaluated the effects of combination of relacorilant and pembrolizumab on advanced ACC and hypercortisolism both on tumoral progression and on hypercortisolism control. The results of this interesting study are expected soon (NCT04373265, www.clinicaltrials.gov). Relacorilant might increase the recruitment and function of natural killer and other immune cells in the tumor microenvironment, promoting immune response in ACC expressing glucocorticoid receptors.

## Immune checkpoint inhibitors and their role in cortisol-secreting ACCs

Immune checkpoint inhibitors (ICIs), such as PD-1, PD-L1, and CTLA-4 blockers, have changed cancer therapy by modulating T-cell inhibitory pathways and enhancing antitumor immunity. Initially approved for malignancies like melanoma, renal cell carcinoma, and non-small-cell lung cancer, ICIs have more recently been investigated in ACC, because PD-L1 expression has been detected on both tumor and infiltrating immune cells [44]. A study analyzing 162 tumor samples from 122 ACC patients revealed that PD-1 was expressed in 26.5%, PD-L1 in 24.7%, and CTLA-4 in 52.5% of cases. Among these, only PD-1 expression was associated with longer progression-free survival [45].

Only a few studies provided data on hormone-secreting ACCs (Table 2). However, cortisol excess may impair immunotherapy efficacy, given that high glucocorticoid exposure (≥ 10 mg prednisone equivalent) is associated with worse outcomes during PD-L1 blockade [46]. Despite the presence of cortisol excess, several studies have reported responses to ICIs in metastatic ACC patients.

**Fig. 4:**
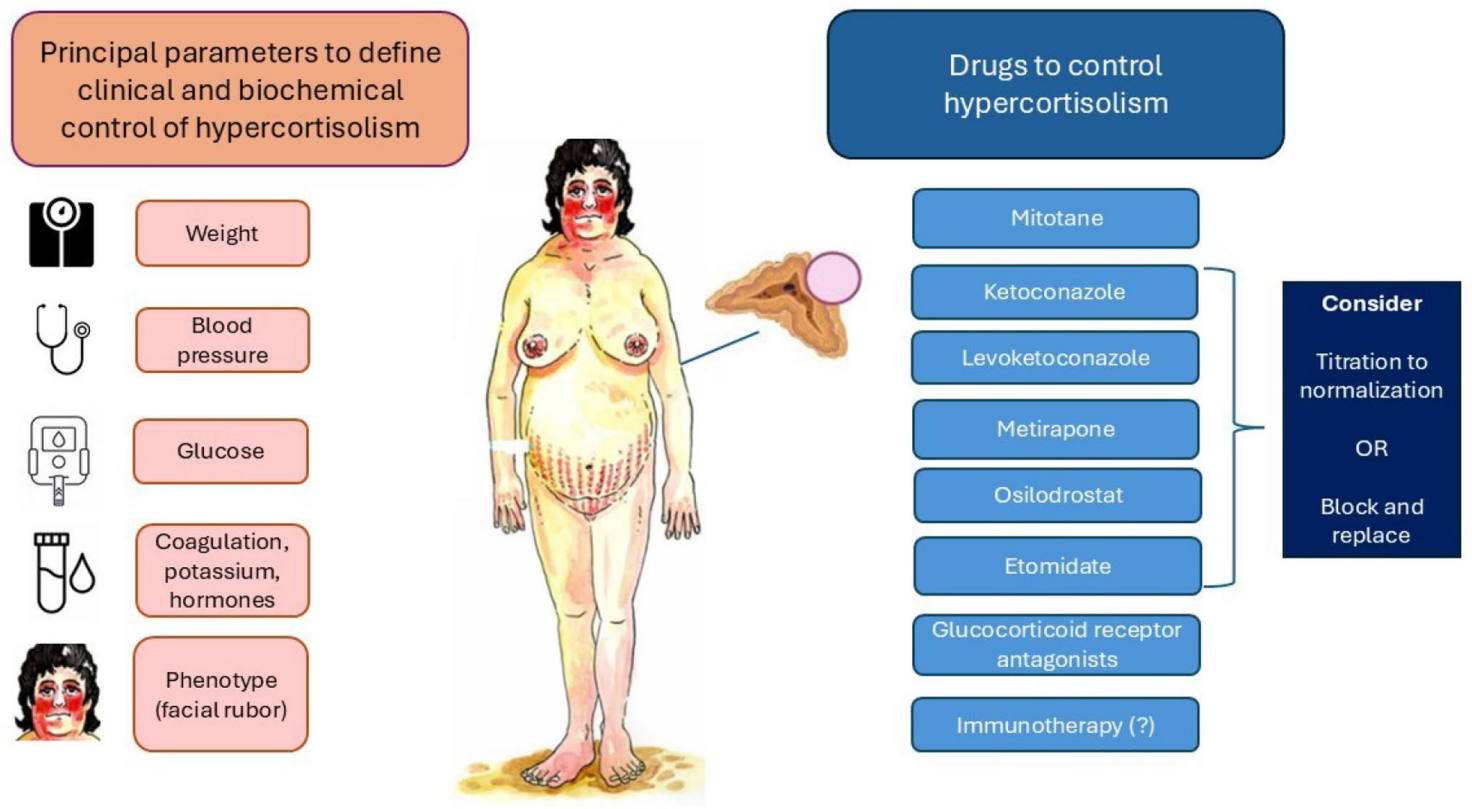
Overview of patients with cortisol-secreting adrenocortical carcinoma, focusing on key clinical and biochemical parameters for assessing hypercortisolism control, as well as the therapeutic options currently available for its management

**Table 2:** List of immune checkpoint inhibitors used in cortisol-secreting ACCs

Authors	Study	Year	Therapy	PDL1-PD1 expression	Patients with cortisol-secreting ACC/Total number of patients	Adrenal insufficiency	Hypercortisolism Control	Combination with mitotane	Tumoral Response
Casey et al.	Case report	2018	Pembrolizumab + mitotane followed by Pembrolizumab + metirapone	< 1%	1	Unknown	Not reported	Yes	PD
Carneiro et al.	Multicentric	2019	Nivolumab	6/10	2/10	1 patient with non-functioning ACC	Not reported	Yes	PD in both patients
Habra et al.	Monocentric Phase 2	2019	Pembrolizumab	0/14	7/16	No	Not reported	No	1 PR 3 SD 3 PD
Head et al.	Retrospective	2019	Pembrolizumab + mitotane	Not reported	3/6	No	Not reported	Yes	3 SD
Bedrose et al.	Retrospective case series	2020	Lenvatinib and pembrolizumab	Not reported	3/8	No	Not reported	No	2 PD 1 SD
Klein et al.	Multicentric open label phase 2	2021	Nivolumab and ipilimumab for 4 doses and after nivolumab	Negative	2/6	1 patient with cortisol-secreting ACC and 1 patient with non-functioning ACC	Not reported	No	1 SD 1 PR
Remde et al.	Multicentric Retrospective	2023	Pembrolizumab (*n* = 32) Nivolumab (*n* = 13) Avelumab (*n* = 6) Atezolizumab (*n* = 1) Ipililumab-nivolumab (*n* = 2)	Positive	22/54	No	Not reported	No	4/22 PR (2 nivolumab, 1 avelumab, 1 pembrolizumab)
Weng et al.	Case report	2023	Sintilimab + etoposide/paraplatin + mitotane	0	1	Yes	Not reported	Yes	PR
Schwarzlmueller et al.	Case series	2024	Pembrolizumab	1 patient: TPS 1%, IC score 0%, CPS 1 2 patient: no expression	3	No	Not reported	No	1 CR 2 PR

Abbreviations: ACC adrenocortical cancer; PD progressive disease, PR partial response; SD stable disease; CR: complete response

Habra et al. observed 1 partial response and 3 stable diseases among 7 cortisol-secreting ACC patients treated with pembrolizumab, without any cases of adrenal insufficiency [47]. Similarly, Head et al. reported initial stability followed by progression in 3 cortisol-secreting ACC patients treated with pembrolizumab and mitotane [48]

Remde et al. found partial responses in 4 out of 22 cortisol-secreting ACC patients treated with various ICIs, although progression was common and data on hypercortisolism control were lacking [49]

Carneiro et al. reported progression in 2 cortisol-secreting ACC patients treated with nivolumab [50]. Interestingly, Bedrose et al. showed that 2 out of 3 cortisol-secreting ACC patients treated with pembrolizumab and lenvatinib, without concomitant mitotane, experienced progressive disease with short progression free survival, while the other had a stable disease [51]. Klein et al. showed that 1 cortisol-secreting ACC patient responded with a partial and complete metabolic response (the patient developed an adrenalitis), while the other had stable disease to nivolumab and ipilimumab [52]

In addition, some single clinical cases and case series on cortisol secreting ACCs have been reported. Casey et al. reported a patient treated with pembrolizumab stopped after 2 weeks due to the liver failure onset [53]. Weng et al. reported one patient treated with sintilimab and chemotherapy (etoposide combined to carboplatin) and mitotane obtaining a long-term stable disease [54]. Schwarzlmueller et al. reported 3 female patients with combined androgen and glucocorticoid hypersecretion ACC syndrome, treated with adrenal surgery, EDP-M, brachytherapy and radiation therapy for distant metastases [55]. Due to the progression of disease, patients were treated with pembrolizumab, at the dose of 200 mg q3w, for 23,15 and 10 cycles, obtaining a complete and a partial remission (2 patients), respectively

The aforementioned studies predominantly address tumoural response while providing limited insights into the management of hypercortisolism. Interestingly, three patients, two with non-functioning ACC and one with cortisol-secreting ACC, treated with nivolumab developed adrenal insufficiency, highlighting a potential impact of this drug on adrenal steroidogenesis. Carneiro et al. reported the case of a patient who developed adrenal insufficiency 18 months after discontinuing high-dose steroid therapy, which had been administered to manage transaminase rise as an adverse effect nivolumab-related, suggesting that this condition may have been mediated by a robust inflammatory response as well as an antitumour effect [50]

Klein at al. reported two patients who developed adrenal insufficiency, one with non-functioning and one with cortisol-secreting ACC, both concurrently treated with mitotane. Interestingly, these two patients were the only responding patients [52]

## Conclusions

Cortisol-secreting ACCs remain a therapeutic challenge for clinicians [55]. They show high aggressiveness and worse prognosis. A rapid control of hypercortisolism should be obtained in all patients to limit the acute complications of glucocorticoid excess. Clinical and biochemical parameters can be used to define hypercortisolism control including blood pressure, weight and clinical picture, potassium, glucose level and coagulation parameters (Fig. 4). Caution should be used for serum, urinary and salivary cortisol determination, because these values could be falsely altered by potential mitotane combined treatment

The therapeutic approach to severe hypercortisolism in ACC involves the use of steroidogenesis inhibitors or glucocorticoid receptor antagonists in combination with mitotane. Drug regimens are highly variable, particularly for metyrapone and ketoconazole, with reported mean doses ranging widely depending on clinical severity and individual response. Metyrapone is highly effective, especially when combined with ketoconazole. Osilodrostat has demonstrated a rapid onset of action, sustained hypocortisolemic effect, and notable efficacy even in cases of severe hypercortisolism.

Cortisol excess has a potential negative impact on immunotherapy efficacy in cortisol-secreting ACC, with only few patients still respond to ICIs. The management of hypercortisolism during ICI therapy remains insufficiently addressed and needs of further research.

Currently there are no evident correlations between hypercortisolism control and improved overall survival in ACC. It may be hypothesized that higher mortality in patients with cortisol-secreting ACCs could be secondary to the detrimental systemic effects of glucocorticoid excess, even though evidence from basic studies suggests that cortisol secretion correlates with a more aggressive molecular signature [56]. Therefore, a rapid and sustained control of hypercortisolism in patients with cortisol-secreting ACCs could improve the high risk of mortality, which is due to both to the tumour and to the effects of cortisol itself.

There is a pressing need for cohort and prospective studies that focus on the effects of rapidly controlling hypercortisolism on the prognosis of patients with ACC. These studies could offer valuable insights into whether early intervention can significantly improve long-term outcomes for these patients. Additionally, further research should explore the differences in overall survival rates between individuals with mild and severe hypercortisolism, as these distinctions may help guide treatment strategies and better tailor interventions to the specific needs of patients.

## Data Availability

No datasets were generated or analysed during the current study.

## Abbreviations

2S,4R: Stereoisomer configuration (e.g., of a drug molecule)
ACCs: Adrenocortical carcinomas
ACTH: Adrenocorticotropic hormone
APC: Adenomatous polyposis coli
CBG: Cortisol binding globulin
CPS: Combined Positive Score
CR: Complete response
CS: Cushing’s syndrome
CTLA-4: Cytotoxic T-lymphocyte-associated protein 4
EDP-M: Etoposide, Doxorubicin, Cisplatin plus Mitotane
EMA: European Medicines Agency
ENSAT: European Network for the Study of Adrenal Tumors
EPCAM: Epithelial cell adhesion molecule
FDA: Food and Drug Administration
HSD11B2: 11-Hydroxysteroid dehydrogenase type 2
ICIs: Immune checkpoint inhibitors
IGF2: Insulin-like growth factor 2
K: Potassium
MEN 1: Multiple endocrine neoplasia type 1
MLH1: MutL homolog 1
MSH2: MutS homolog 2
MSH6: MutS homolog 6
PD: Progressive disease
PD-1: Programmed cell death protein 1
PD-L1: Programmed death-ligand 1
PMS2: Postmeiotic segregation increased 2
PR: Partial response
PRKAR1A: Protein kinase cAMP-dependent type I regulatory subunit alpha
Pts: Patients
QID: Quarter in die (four times a day)
SD: Stable disease
TID: Ter in die (three times a day)
TP53: Tumor protein p53 gene
TPS: Tumor Proportion Score
UFC: Urinary free cortisol
US: United States
H: Hour
Kg: Kilograms
Mg: Milligrams
q3w: Every 3 weeks (quaque 3 weeks)
S: Seconds
